# Supplementary material for: Analysing the impact of modifiable risk factors on cardiovascular disease mortality in Brazil
Source: PLoS One. 2022 Jun 22;17(6):e0269549. doi: 10.1371/journal.pone.0269549 (PMC9216570; doi:10.1371/journal.pone.0269549)
Supplement: S1 Table — (DOCX) [file pone.0269549.s001.docx]

## Supplementary Table 1: Descriptive information on variables and data sources used across 26 Brazilian states from 2005 to 2017.

| **Variable name** | **Unit** | **Source** | **Date last accessed** |
| --- | --- | --- | --- |
| **Socioeconomic indicators and care services** | | | |
| GDP per capita | Reais (R$) | IBGE. Gross domestic product at current prices, taxes, net of subsidies, on products at current prices and gross value added at current prices, total and by economic activity, and respective shares – Reference year of 2010. Available at: https://sidra.ibge.gov.br/tabela/5938 | 14^th^ July 2021 |
| Gini Index | N/A | IBGE. Gini Index of the gross domestic product at current prices – Reference year of 2010. Available at: https://sidra.ibge.gov.br/Tabela/5939 | 14^th^ July 2021 |
| Bolsa Família | Reais (R$) | Brazilian Ministry of Social Development. Available at: https://aplicacoes.mds.gov.br/sagi/vis/data3/data-explorer.php | 23^rd^ Jun 2021 |
| Hospital beds | Beds per 1,000 inhabitants | Brazilian Ministry of Health /SAS – National Registry of Health Facilities (CNES). Available at: http://tabnet.datasus.gov.br/cgi/deftohtm.exe?cnes/cnv/leiintbr.def | 14^th^ July 2021 |
| Coverage of primary care | % of people | Brazilian Ministry of Health – National Health Agency. Available at: https://bit.ly/2SQBSWf | 14^th^ July 2021 |
| **Health outcomes and risks** | | | |
| Mortality rate | Deaths per 100,000.00 | Global Health Data Exchange/GBD Results Tool. Available at: http://ghdx.healthdata.org/gbd-results-tool | 21^st^ September 2021 |
| Years of life lost (YLL) | Days | Global Health Data Exchange/GBD Results Tool. Available at: http://ghdx.healthdata.org/gbd-results-tool | 21^st^ September 2021 |
| Summary Exposure value (SEV), risk factors | Risk-adjusted prevalence, from 0 to 1 | Global Health Data Exchange/GBD Results Tool. Available at: http://ghdx.healthdata.org/gbd-results-tool | 21^st^ September 2021 |
| Population attributable fraction (PAF) | % | Global Health Data Exchange/GBD Compare Tool. Available at: https://vizhub.healthdata.org/gbd-compare/ | 23^rd^ September 2021 |
