# Supplementary material for: Analysing the impact of modifiable risk factors on cardiovascular disease mortality in Brazil
Source: PLoS One. 2022 Jun 22;17(6):e0269549. doi: 10.1371/journal.pone.0269549 (PMC9216570; doi:10.1371/journal.pone.0269549)
Supplement: S2 Table — (DOCX) [file pone.0269549.s002.docx]

## Supplementary Table 2: International Classification of Diseases (ICD) codes and hierarchy for non-communicable diseases (NCDs) included in the analysis.

| **Metric** | **ICD-10** | **ICD-9** | **Hierarchy** |
| --- | --- | --- | --- |
| Cardiovascular diseases | B33·2, G45-G46·8, I01-I01·9, I02·0, I05-I09·9, I11-I11·9, I20-I25·9, I28-I28·8, I30-I31·1, I31·8-I37·8, I38-I41·9, I42·1-I42·8, I43-I43·9, I47-I48·9, I51·0-I51·4, I60-I63·9, I65-I66·9, I67·0-I67·3, I67·5-I67·6, I68·0-I68·2, I69·0-I69·3, I70·2-I70·8, I71-I73·9, I77-I83·9, I86-I89·0, I89·9, I98, K75·1 | 036·4, 391-391·9, 392·0, 393-398·9, 402-402·9, 410-414·9, 417-417·9, 420-423, 423·1-423·9, 424·0-424·9, 425·0-425·3, 425·5, 425·7-425·8, 427-427·3, 427·6-427·8, 429·0, 430-435·9, 437·0-437·2, 437·5-437·8, 440·2, 440·4, 441-443·9, 447-454·9, 456, 456·3-457, 457·1, 457·8-457·9, 459, 459·1-459·3 | DE 2 |
| Ischaemic heart disease | I20-I25·9 | 410-414·9 | DE 3 |
| Ischaemic stroke | G45-G46·8, I63-I63·9, I65-I66·9, I67·2-I67·3, I67·5-I67·6, I69·3 | 433-435·9, 437·0-437·1, 437·5-437·8 | DE 4 |
| Diabetes mellitus type 2 | E11-E11·1, E11·3-E11·9 |  | DE 4 |
|  |  |  |  |

ICD is the global standard for health data, clinical documentation, and statistical aggregation. Abbreviations: DE= Disease endpoint
