# Supplementary material for: Analysing the impact of modifiable risk factors on cardiovascular disease mortality in Brazil
Source: PLoS One. 2022 Jun 22;17(6):e0269549. doi: 10.1371/journal.pone.0269549 (PMC9216570; doi:10.1371/journal.pone.0269549)
Supplement: S3 Table — (DOCX) [file pone.0269549.s003.docx]

## Supplementary Table 3: Metadata of risk factors used.

| **Metric** | **Exposure definition** | **Theoretical minimum exposure level** | **Hierarchy** |
| --- | --- | --- | --- |
| **Metabolic risks** |  |  | RF 1 |
| High fasting plasma glucose | Serum fasting plasma glucose, measured in mmol/L | 4.8–5.4 mmol/L | RF 2 |
| High systolic blood pressure | Systolic blood pressure, measured in mm Hg | 110–115 mm Hg | RF 2 |
| High body-mass index | Body-mass index, measured in kg/m² | 20–25 kg/m² | RF 2 |
| **Behavioural risks** |  |  | RF 1 |
| Smoking | Prevalence of current use of any smoked tobacco product and prevalence of former use of any smoked tobacco product; among current smokers, cigarette equivalents smoked per smoker per day and cumulative pack-years of exposure; among former smokers, number of years since quitting | All individuals are lifelong non-smokers | RF 3 |

RF= Risk factor
