# Supplementary material for: Analysing the impact of modifiable risk factors on cardiovascular disease mortality in Brazil
Source: PLoS One. 2022 Jun 22;17(6):e0269549. doi: 10.1371/journal.pone.0269549 (PMC9216570; doi:10.1371/journal.pone.0269549)
Supplement: S4 Table — (DOCX) [file pone.0269549.s004.docx]

## Supplementary Table 4: Glossary of metrics.

| **Metric** | **Definition** |
| --- | --- |
| Summary Exposure value (SEV) | A measure of a population’s exposure to a risk factor that takes into account the extent of exposure by risk level and the severity of that risk’s contribution to disease burden. SEV takes the value zero when no excess risk for a population exists and the value one when the total population is at the highest level of risk; we report SEV on a scale from 0% to 100% to emphasize that it is risk-weighted prevalence. |
| Year of Life Lost (YLL) | Years of life lost due to premature mortality. YLLs are the multiplication of deaths and a standard life expectancy at the age of death. The standard life expectancy is derived from a life table that contains the lowest observed mortality rate at each age that has been observed in any population greater than 5 million. |
| Population attributable fraction (PAF) | The population attributable fraction is the proportional reduction of mortality that would occur in a population if the exposure to a risk factor were reduced to an alternative ideal exposure scenario. The number of deaths attributed to a risk factor is quantified by applying the population attributable fraction to the total number of deaths. |
| Primary health | Primary Health Care (PHC) is the first level of health care and is characterized by a set of health actions, at the individual and collective levels, covering health promotion and protection, disease prevention, diagnosis, treatment, rehabilitation, harm reduction and health maintenance in order to develop comprehensive care that has a positive impact on the health situation of communities. |
| GDP per capita | GDP per capita is gross domestic product divided by midyear population. GDP is the sum of gross value added by all resident producers in the economy plus any product taxes and minus any subsidies not included in the value of the products. It is calculated without making deductions for depreciation of fabricated assets or for depletion and degradation of natural resources. |
| Gini Index | Gini index measures the extent to which the distribution of income (or, in some cases, consumption expenditure) among individuals or households within an economy deviates from a perfectly equal distribution. A Lorenz curve plots the cumulative percentages of total income received against the cumulative number of recipients, starting with the poorest individual or household. The Gini index measures the area between the Lorenz curve and a hypothetical line of absolute equality, expressed as a percentage of the maximum area under the line. Thus, a Gini index of 0 represents perfect equality, while an index of 100 implies perfect inequality. |
