# Supplementary material for: Analysing the impact of modifiable risk factors on cardiovascular disease mortality in Brazil
Source: PLoS One. 2022 Jun 22;17(6):e0269549. doi: 10.1371/journal.pone.0269549 (PMC9216570; doi:10.1371/journal.pone.0269549)
Supplement: S5 Table — (DOCX) [file pone.0269549.s005.docx]

## Supplementary Table 5: Descriptive information of the summary exposure value (SEV) of risk factors^a^ in 2005 and 2017 in the 26 Brazilian states.

| **State** | **Mortality by cardiovascular diseases^b^** | | **SEV high glucose** | | **SEV high blood pressure** | | **SEV high low-density lipoprotein** | | **SEV high body mass index** | | **SEV smoking** | |
| --- | --- | --- | --- | --- | --- | --- | --- | --- | --- | --- | --- | --- |
|  | 2005 | 2017 | 2005 | 2017 | 2005 | 2017 | 2005 | 2017 | 2005 | 2017 | 2005 | 2017 |
| **North region** |  |  |  |  |  |  |  |  |  |  |  |  |
| Acre | 206.21 | 173.95 | 10.65 | 12.27 | 22.52 | 22.93 | 41.66 | 44.08 | 21.28 | 29.68 | 16.67 | 11.49 |
| Amapá | 160.03 | 155.60 | 9.60 | 11.72 | 21.57 | 21.86 | 43.44 | 45.42 | 25.54 | 32.23 | 11.90 | 8.57 |
| Amazonas | 178.50 | 144.72 | 10.07 | 12.11 | 19.73 | 20.07 | 42.56 | 44.75 | 23.24 | 30.78 | 11.31 | 8.00 |
| Pará | 201.17 | 164.12 | 10.45 | 12.33 | 21.89 | 22.32 | 41.74 | 43.96 | 20.19 | 28.31 | 11.81 | 8.03 |
| Rondônia | 262.94 | 177.76 | 12.33 | 12.58 | 22.40 | 23.06 | 42.50 | 45.05 | 22.21 | 31.70 | 14.52 | 9.91 |
| Roraima | 254.03 | 184.51 | 13.28 | 14.49 | 22.20 | 22.68 | 42.19 | 44.31 | 23.25 | 31.25 | 14.68 | 9.88 |
| Tocantins | 219.09 | 204.82 | 10.27 | 12.20 | 16.87 | 17.39 | 41.15 | 44.10 | 18.59 | 28.48 | 12.52 | 8.87 |
| **Northeast region** |  |  |  |  |  |  |  |  |  |  |  |  |
| Alagoas | 250.51 | 230.79 | 13.27 | 15.43 | 26.57 | 26.96 | 41.14 | 43.20 | 19.54 | 27.10 | 12.07 | 7.85 |
| Bahia | 213.02 | 175.07 | 10.67 | 12.51 | 27.90 | 28.52 | 41.90 | 43.94 | 20.08 | 27.46 | 10.92 | 7.24 |
| Ceará | 207.99 | 188.00 | 9.66 | 11.71 | 24.41 | 24.78 | 42.03 | 44.11 | 20.55 | 28.35 | 13.26 | 8.99 |
| Maranhão | 208.91 | 243.91 | 11.38 | 13.20 | 21.75 | 22.30 | 39.46 | 41.67 | 15.32 | 23.17 | 10.23 | 7.07 |
| Paraíba | 219.62 | 187.36 | 12.02 | 13.80 | 23.88 | 24.36 | 41.35 | 43.57 | 19.22 | 26.57 | 12.67 | 8.67 |
| Pernambuco | 245.74 | 229.22 | 12.03 | 13.63 | 25.41 | 25.83 | 42.27 | 44.45 | 20.31 | 27.86 | 13.06 | 8.91 |
| Piauí | 228.90 | 177.00 | 10.23 | 12.58 | 21.81 | 22.20 | 40.31 | 42.40 | 16.69 | 24.57 | 12.34 | 8.78 |
| Rio Grande do Norte | 186.22 | 154.10 | 8.99 | 13.31 | 24.27 | 24.66 | 42.12 | 44.54 | 21.26 | 29.54 | 12.24 | 8.28 |
| Sergipe | 193.70 | 171.77 | 12.55 | 13.90 | 28.70 | 29.28 | 42.40 | 44.49 | 20.80 | 28.40 | 10.52 | 7.26 |
| **Central-west region** |  |  |  |  |  |  |  |  |  |  |  |  |
| Goiás | 240.37 | 171.14 | 9.80 | 11.09 | 24.66 | 25.04 | 42.89 | 45.19 | 22.38 | 30.88 | 14.81 | 9.48 |
| Mato Grosso | 224.83 | 157.74 | 10.51 | 12.06 | 21.52 | 22.11 | 43.48 | 45.98 | 25.11 | 34.73 | 14.32 | 9.46 |
| Mato Grosso do Sul | 256.56 | 183.65 | 12.10 | 12.83 | 28.78 | 29.26 | 44.03 | 46.38 | 24.89 | 33.29 | 14.79 | 9.78 |
| **Southeast region** |  |  |  |  |  |  |  |  |  |  |  |  |
| Espírito Santo | 244.93 | 196.55 | 10.22 | 11.46 | 24.44 | 24.99 | 43.54 | 45.80 | 22.90 | 31.53 | 13.84 | 8.81 |
| Minas Gerais | 217.27 | 148.49 | 8.14 | 9.72 | 25.83 | 25.76 | 45.46 | 48.83 | 21.17 | 28.98 | 14.76 | 10.34 |
| Rio de Janeiro | 255.38 | 197.96 | 11.48 | 12.21 | 28.41 | 28.95 | 40.97 | 42.97 | 26.56 | 33.25 | 14.34 | 9.51 |
| São Paulo | 238.71 | 178.01 | 11.22 | 10.85 | 29.61 | 29.90 | 42.35 | 44.33 | 26.63 | 33.56 | 16.74 | 11.63 |
| **South region** |  |  |  |  |  |  |  |  |  |  |  |  |
| Paraná | 245.54 | 181.62 | 10.41 | 11.72 | 29.01 | 29.49 | 44.23 | 46.17 | 24.95 | 32.35 | 17.55 | 11.31 |
| Rio Grande do Sul | 226.19 | 168.38 | 8.99 | 10.12 | 30.01 | 30.09 | 40.62 | 42.43 | 27.85 | 34.26 | 20.43 | 12.88 |
| Santa Catarina | 238.99 | 165.75 | 9.91 | 10.83 | 37.38 | 32.90 | 44.33 | 46.29 | 26.20 | 34.43 | 16.62 | 10.77 |

^a^ Age-standardized SEV displayed for both sexes per 100.000 people. ^b^ Age-standardized mortality displayed for both sexes per 100.000 people.
