# Supplementary material for: Analysing the impact of modifiable risk factors on cardiovascular disease mortality in Brazil
Source: PLoS One. 2022 Jun 22;17(6):e0269549. doi: 10.1371/journal.pone.0269549 (PMC9216570; doi:10.1371/journal.pone.0269549)
Supplement: S6 Table — (DOCX) [file pone.0269549.s006.docx]

## Supplementary Table 6: Descriptive information on socioeconomic indicators and care services: GDP per capita, Gini index, bolsa família investment, hospital beds, and coverage of primary healthcare rate in 2005 and 2017 in the 26 Brazilian states.

| **State** | **GDP per capita^a^**  (R$ x 1.000) | | **Gini index^b^** | | **Bolsa Família Investment**  **(**R$ x 100 mi**)** | | **Hospital beds**  **(**per 1.000**)** | | **Primary healthcare**  (% population) | |
| --- | --- | --- | --- | --- | --- | --- | --- | --- | --- | --- |
|  | 2005 | 2017 | 2005 | 2017 | 2005 | 2017 | 2005 | 2017 | 2005 | 2017 |
| **North region** |  |  |  |  |  |  |  |  |  |  |
| Acre | 6.53 | 17.11 | 0.73 | 0.68 | 0.26 | 2.60 | 2.07 | 1.65 | 40.90 | 17.20 |
| Amapá | 7.23 | 19.30 | 0.78 | 0.76 | 0.09 | 1.67 | 1.63 | 1.10 | 32.18 | 30.07 |
| Amazonas | 10.43 | 24.72 | 0.90 | 0.86 | 1.03 | 10.15 | 1.78 | 1.41 | 33.72 | 32.60 |
| Pará | 5.77 | 18.75 | 0.73 | 0.71 | 2.41 | 6.54 | 1.84 | 1.71 | 23.23 | 38.14 |
| Rondônia | 8.17 | 25.35 | 0.66 | 0.69 | 0.45 | 1.54 | 2.38 | 2.46 | 28.07 | 42.06 |
| Roraima | 8.13 | 26.48 | 0.74 | 0.73 | 0.12 | 1.12 | 1.46 | 1.66 | 36.51 | 13.97 |
| Tocantins | 5.87 | 22.58 | 0.69 | 0.72 | 0.49 | 2.7 | 1.83 | 1.67 | 26.62 | 11.91 |
| **Northeast region** |  |  |  |  |  |  |  |  |  |  |
| Alagoas | 5.01 | 16.10 | 0.72 | 0.72 | 1.87 | 8.80 | 2.01 | 1.80 | 12.16 | 11.54 |
| Bahia | 6.22 | 18.35 | 0.79 | 0.78 | 7.51 | 38.63 | 2.21 | 1.87 | 30.31 | 22.21 |
| Ceará | 5.05 | 16.50 | 0.79 | 0.78 | 5.19 | 21.81 | 2.16 | 1.84 | 42.62 | 16.98 |
| Maranhão | 4.05 | 12.95 | 0.74 | 0.72 | 3.73 | 24.12 | 2.66 | 1.78 | 31.75 | 13.33 |
| Paraíba | 4.80 | 15.80 | 0.76 | 0.78 | 2.40 | 11.80 | 2.87 | 2.05 | 9.88 | 4.57 |
| Pernambuco | 5.87 | 19.51 | 0.79 | 0.79 | 4.42 | 23.74 | 2.51 | 2.13 | 42.25 | 22.52 |
| Piauí | 3.52 | 13.96 | 0.76 | 0.76 | 2.01 | 10.73 | 2.64 | 2.12 | 15.06 | 2.11 |
| Rio Grande do Norte | 6.50 | 18.66 | 0.80 | 0.79 | 1.62 | 7.19 | 2.62 | 2.06 | 15.88 | 4.94 |
| Sergipe | 7.29 | 18.20 | 0.73 | 0.73 | 1.03 | 5.41 | 2.04 | 1.34 | 35.79 | 15.93 |
| **Central-west region** |  |  |  |  |  |  |  |  |  |  |
| Goiás | 9.54 | 28.32 | 0.78 | 0.78 | 1.06 | 5.58 | 3.16 | 2.58 | 27.39 | 21.86 |
| Mato Grosso | 12.27 | 38.32 | 0.71 | 0.70 | 0.68 | 3.07 | 2.43 | 2.05 | 38.63 | 32.75 |
| Mato Grosso do Sul | 10.41 | 36.88 | 0.67 | 0.68 | 0.40 | 2.56 | 2.75 | 2.02 | 41.65 | 35.03 |
| **Southeast region** |  |  |  |  |  |  |  |  |  |  |
| Espírito Santo | 13.61 | 29.07 | 0.79 | 0.74 | 0.98 | 3.23 | 2.17 | 1.94 | 27.27 | 29.45 |
| Minas Gerais | 9.78 | 27.65 | 0.82 | 0.81 | 6.18 | 20.76 | 2.41 | 1.93 | 35.38 | 18.53 |
| Rio de Janeiro | 17.44 | 39.57 | 0.84 | 0.83 | 1.80 | 16.06 | 3.13 | 2.17 | 28.61 | 31.47 |
| São Paulo | 18.67 | 47.83 | 0.87 | 0.87 | 5.22 | 28.06 | 2.39 | 2.06 | 19.62 | 30.62 |
| **South region** |  |  |  |  |  |  |  |  |  |  |
| Paraná | 12.58 | 37.88 | 0.79 | 0.77 | 2.44 | 21.72 | 2.88 | 2.44 | 30.10 | 29.57 |
| Rio Grande do Sul | 12.77 | 37.70 | 0.81 | 0.78 | 2.34 | 6.95 | 2.81 | 2.75 | 17.03 | 21.12 |
| Santa Catarina | 13.82 | 39.88 | 0.75 | 0.76 | 0.80 | 2.28 | 2.58 | 2.19 | 23.48 | 17.20 |

^a^GDP per capita is the state GDP in R$ divided by its population. ^b^Gini index is a measure of inequality based on household income. It ranges between 0 and 1, where 0 indicates total equality and 1 indicates total inequality
