# Supplementary material for: Analysing the impact of modifiable risk factors on cardiovascular disease mortality in Brazil
Source: PLoS One. 2022 Jun 22;17(6):e0269549. doi: 10.1371/journal.pone.0269549 (PMC9216570; doi:10.1371/journal.pone.0269549)
Supplement: S7 Table — (DOCX) [file pone.0269549.s007.docx]

## Supplementary Table 7. Stepwise addition of variables used in the main model

|  | **Model 1** | **Model 2** | **Model 3** | **Model 4** |
| --- | --- | --- | --- | --- |
|  | *Coefficient (95% CI)* | *Coefficient (95% CI)* | *Coefficient (95% CI)* | *Coefficient (95% CI)* |
| **SEV high glucose** | **6.79 (2.05 to 11.53)**** | **8.09 (2.30 to 13.88)**** | **8.93 (4.41 to 13.46)***** | **9.71 (6.32 to 13.10)***** |
| **Gini Index** |  | -31.85 (-176.75 to 113.04) | -36.83 (-133.51 to 59.83) | -69.56 (-152.64 to 13.51) |
| **lnGDP per capita** |  | **16.69 (3.15 to 30.24)*** | 10.39 (-1.73 to 22.53) | **35.11 (9.64 to 60.58)**** |
| **logBolsa familia** |  | 3.28 (-10.88 to 17.45) | 0.98 (-7.43 to 9.39) | 0.53 (-6.19 to 7.26) |
| **Hospital beds** |  |  | **26.54 (16.32 to 36.75)***** | **18.73 (5.45 to 32.01)**** |
| **Coverage primary care** |  |  | -0.14 (-0.77 to 0.49) | -0.07 (-0.68 to 0.53) |
| **SEV high BMI** |  |  |  | **-4.72 (-7.99 to -1.46)**** |
| **SEV high LDL** |  |  |  | -0.22 (-2.21 to 1.76) |
| **SEV high SBP** |  |  |  | 0.99 (-0.07 to 2.05) |
| **SEV Smoking** |  |  |  | 2.70 (-1.95 to 7.37) |

Data on mortality by cardiovascular diseases in women were used in this analysis. All models employed state and time fixed effects. * p<0.05; ** p<0.01; *** p<0.001. SEV: summary exposure value. GDP: gross domestic product. BMI: body mass index. LDL: low-density lipoprotein. SBP: systolic blood pressure.

Model 1: SEV high glucose.

Model 2: SEV high glucose and socioeconomic variables (Gini Index, GDP per capita and Bolsa Família investment).

Model 3: SEV high glucose, socioeconomic variables and access to healthcare (hospital beds and coverage of primary care).

Model 4: SEV high glucose, socioeconomic variables, access to healthcare and other risk factors (SEV high BMI, SEV high LDL, SEV high SBP, SEV Smoking).
