# Supplementary material for: Analysing the impact of modifiable risk factors on cardiovascular disease mortality in Brazil
Source: PLoS One. 2022 Jun 22;17(6):e0269549. doi: 10.1371/journal.pone.0269549 (PMC9216570; doi:10.1371/journal.pone.0269549)
Supplement: S8 Table — (DOCX) [file pone.0269549.s008.docx]

## Supplementary Table 8. Association between the prevalence of diabetes and mortality by cardiovascular diseases in men and women, Brazil, 2005 to 2017.

|  | **Men** | | | **Women** | | |
| --- | --- | --- | --- | --- | --- | --- |
|  | **Cardiovascular Diseases**  (Coefficient, 95%CI) | **Ischaemic heart disease**  (Coefficient, 95%CI) | **Ischaemic stroke**  (Coefficient, 95%CI) | **Cardiovascular Diseases**  (Coefficient, 95%CI) | **Ischaemic heart disease**  (Coefficient, 95%CI) | **Ischaemic stroke**  (Coefficient, 95%CI) |
| **Prevalence of Diabetes** | **0.02 (0.01 to 0.04)**** | **0.001 (0.0006 to 0.02)***** | 0.003 (-0.0001 to 0.008) | **0.002 (0.01 to 0.03)***** | **0.008 (0.0005 to 0.01)***** | **0.005 (0.002 to 0.008)***** |
| **SEV high BMI** | **-9.07 (-14.65 to -3.48)**** | **-3.14 (-5.60 to -0.68)*** | **-2.11 (-3.78 to -0.45)*** | **-4.69 (-7.94 to -1.44)**** | **-1.50 (-2.91 to -0.08)*** | -1.17 (-2.35 to 0.004) |
| **SEV high LDL** | -3.12 (-7.42 to 1.18) | -0.81 (-3.01 to 1.38) | **-2.13 (-3.61 to -0.65)**** | 4.39 (-1.89 to 1.98) | -0.24 (-1.06 to 0.57) | -0.06 (-1.07 to 0.93) |
| **SEV high SBP** | -0.55 (-3.09 to 1.99) | -0.18 (-1.31 to 0.94) | -0.004 (-0.78 to 0.77) | 0.94 (-0.12 to 2.00) | **0.80 (0.42 to 1.18)***** | 0.21 (-0.16 to 0.58) |
| **SEV Smoking** | **6.72 (1.25 to 12.20)*** | 2.45 (-0.38 to 5.29) | 1.57 (-3.98 to 3.54) | 3.05 (-1.60 to 7.72) | 0.75 (-1.18 to 2.69) | 1.37 (-0.10 to 2.85) |

The full model adjusted for Gini Index, GDP per capita, Bolsa Família investment, hospital beds, coverage of primary care and state and time fixed effects. * p<0.05; ** p<0.01; *** p<0.001. SEV: summary exposure value. GDP: gross domestic product. BMI: body mass index. LDL: low-density lipoprotein. SBP: systolic blood pressure.
