# Supplementary material for: Analysing the impact of modifiable risk factors on cardiovascular disease mortality in Brazil
Source: PLoS One. 2022 Jun 22;17(6):e0269549. doi: 10.1371/journal.pone.0269549 (PMC9216570; doi:10.1371/journal.pone.0269549)
Supplement: S9 Table — (DOCX) [file pone.0269549.s009.docx]

## Supplementary Table 9. Lag-time analysis between the prevalence of diabetes and the mortality by cardiovascular diseases in men and women, Brazil, 2005 to 2017.

|  | **Men** | | | | **Women** | | | |
| --- | --- | --- | --- | --- | --- | --- | --- | --- |
|  | **2-year lag**  (Coefficient, 95%CI) | **5-year lag**  (Coefficient, 95%CI) | **8-year lag**  (Coefficient, 95%CI) | **10-year lag**  (Coefficient, 95%CI) | **2-year lag**  (Coefficient, 95%CI) | **5-year lag**  (Coefficient, 95%CI) | **8-year lag**  (Coefficient, 95%CI) | **10-year lag**  (Coefficient, 95%CI) |
| **Prevalence of Diabetes** | **0.029**  **(0.011 to 0.048)**** | **0.027**  **(0.007 to 0.047)**** | **0.026**  **(0.004 to 0.048)*** | **0.027**  **(0.001 to 0.052)*** | **0.022**  **(0.014 to 0.030)***** | **0.017**  **(0.009 to 0.025)***** | **0.017**  **(0.008 to 0.026)***** | **0.017**  **(0.008 to 0,027)***** |

The full model was adjusted for Gini Index, GDP per capita, Bolsa Família investment, hospital beds, coverage of primary care, SEV of high BMI, SEV of high LDL, SEV of high SBP, SEV of smoking and state and time fixed effects. * p<0.05; ** p<0.01; *** p<0.001. SEV: summary exposure value. GDP: gross domestic product. BMI: body mass index. LDL: low-density lipoprotein. SBP: systolic blood pressure.
