# Supplementary figures and images for: Analysing the impact of modifiable risk factors on cardiovascular disease mortality in Brazil
Source: PLoS One. 2022 Jun 22;17(6):e0269549. doi: 10.1371/journal.pone.0269549 (PMC9216570; doi:10.1371/journal.pone.0269549)

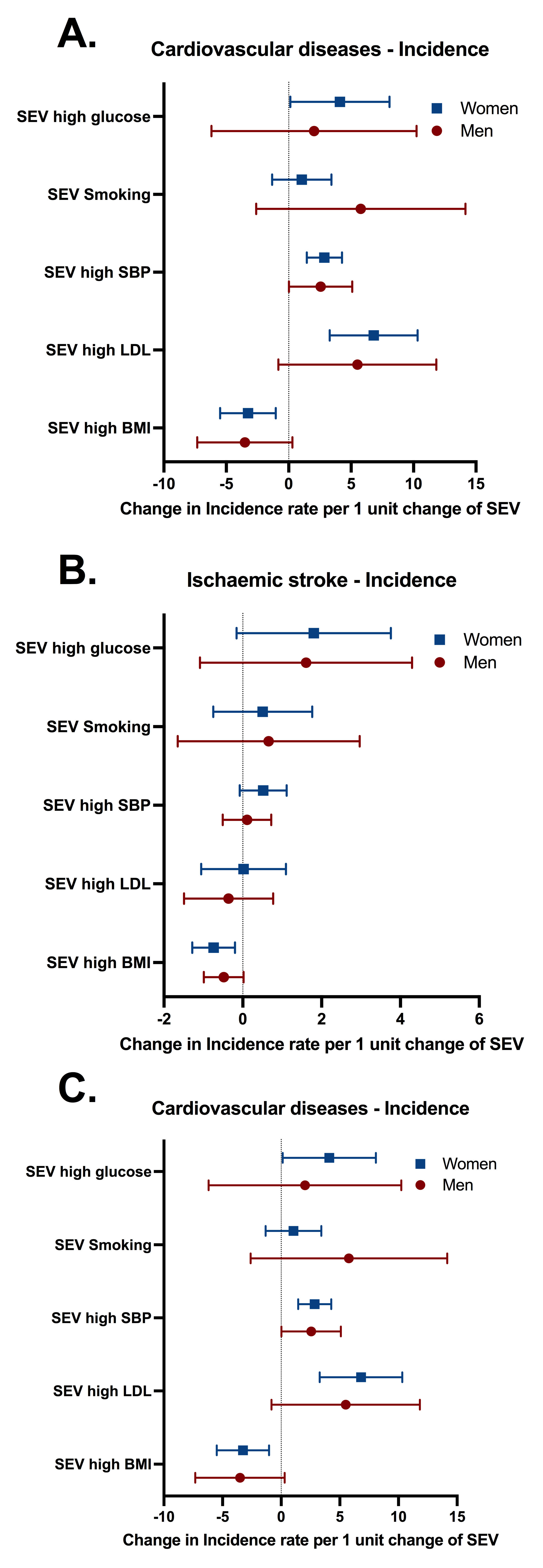

Supplement: S1 Fig — Associations between SEV for risk factors and incidence rates for (A) cardiovascular diseases, (B) ischaemic stroke, and (C) ischaemic heart disease for men and women in Brazil. Variables included in the model: doctors per 1,000 habitants, hospital beds per 1,000 habitants, coverage of primary care, Bolsa Família transfer, GDP per capita, state and year fixed effects and SEV of risk factors. Incidence and SEV were per 100,000 people. Estimates and 95% confidence intervals (CI) are provided in the figure. SBP: systolic blood pressure. LDL: low-density lipoprotein. BMI: body mass index. (TIFF) [file pone.0269549.s010.tiff]

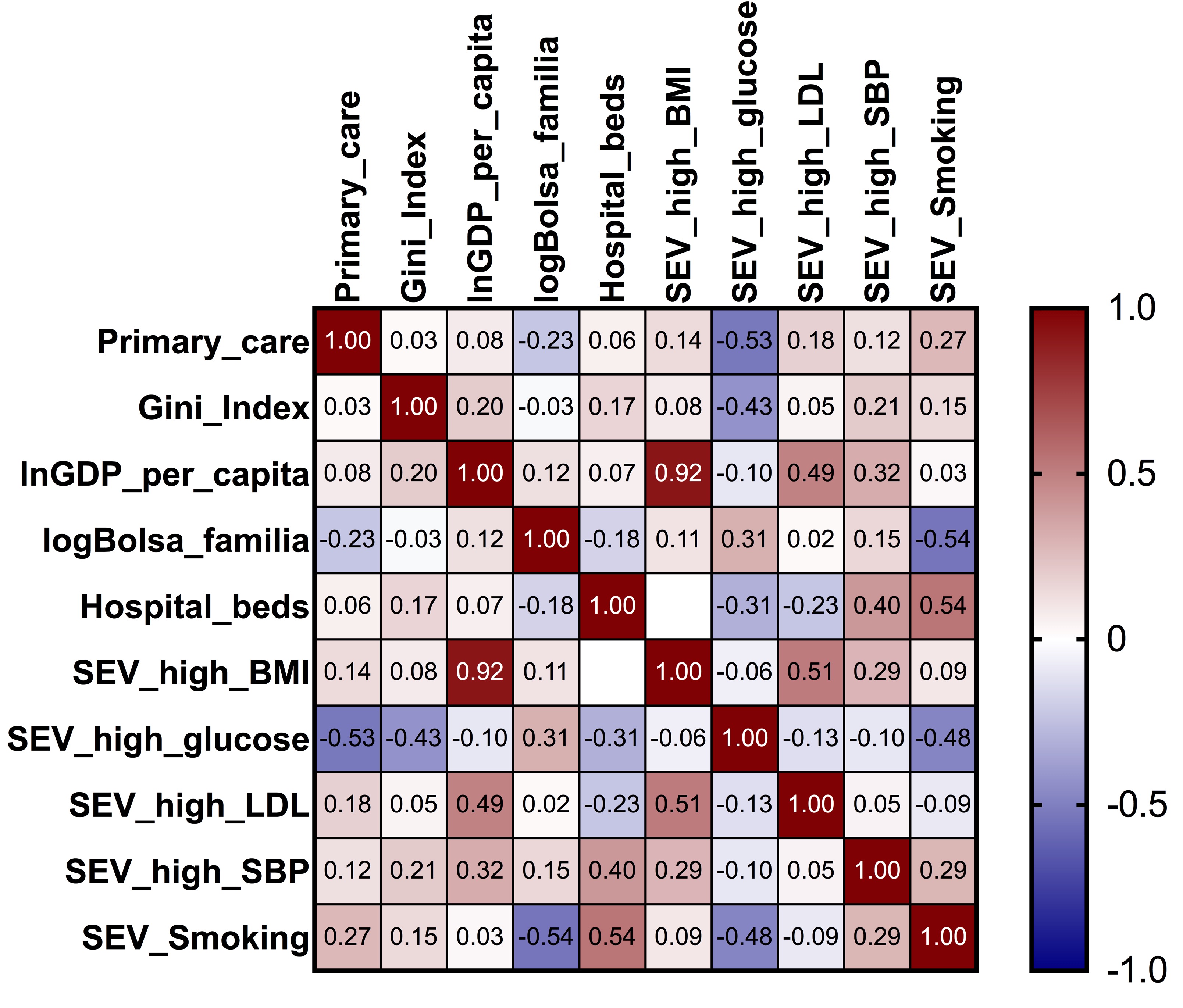

Supplement: S2 Fig — The Pearson r is shown in cells for correlations between individual variables. Correlations > 0.7 or <-0.7 were considered indicative of autocorrelation. GDP: growth domestic product. SEV: summary exposure value. BMI: body mass index. LDL: low-density lipoprotein. SBP: systolic blood pressure. Correlations reported for females. (JPG) [file pone.0269549.s011.jpg]

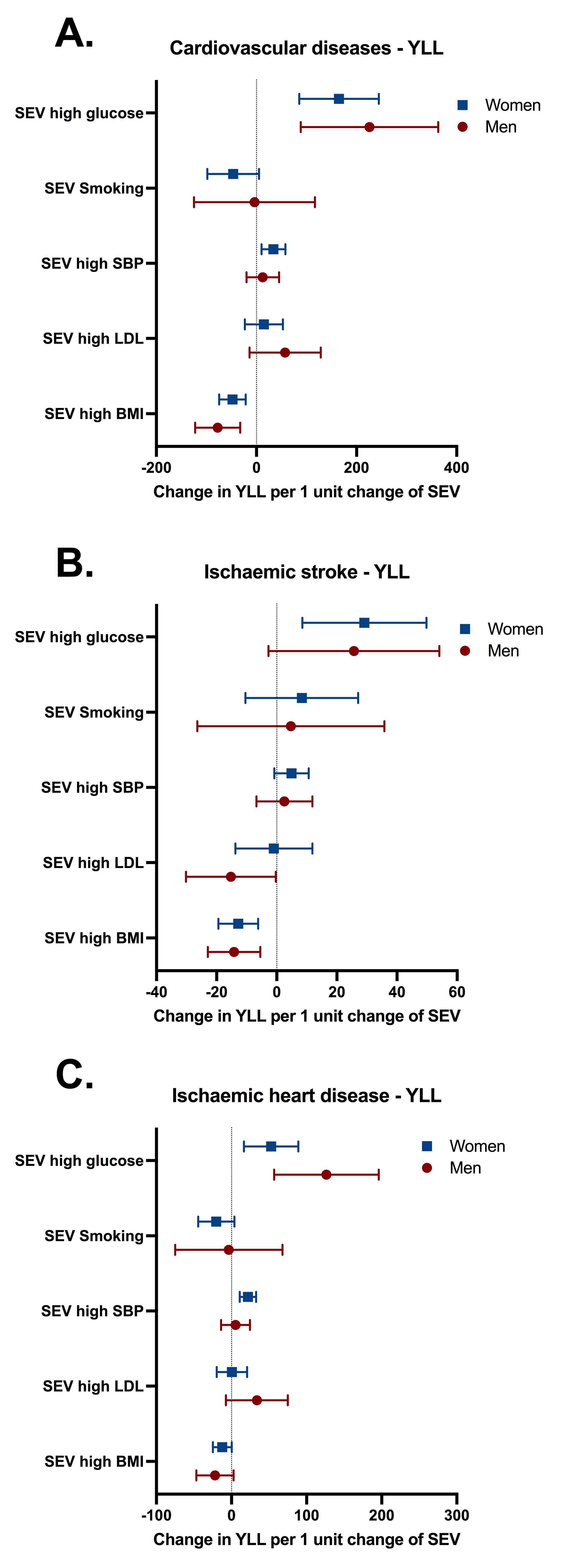

Supplement: S3 Fig — Associations between SEV for risk factors and years of life lost (YLL) for (A) cardiovascular diseases, (B) ischaemic stroke, and (C) ischaemic heart disease for men and women in Brazil. Variables included in the model: doctors per 1,000 habitants, hospital beds per 1,000 habitants, coverage of primary care, Bolsa Família transfer, GDP per capita, state and year fixed effects and SEV of risk factors. Mortality and SEV were per 100,000 people. Estimates and 95% confidence intervals (CI) are provided in the figure. SBP: systolic blood pressure. LDL: low-density lipoprotein. BMI: body mass index. (TIFF) [file pone.0269549.s012.tiff]

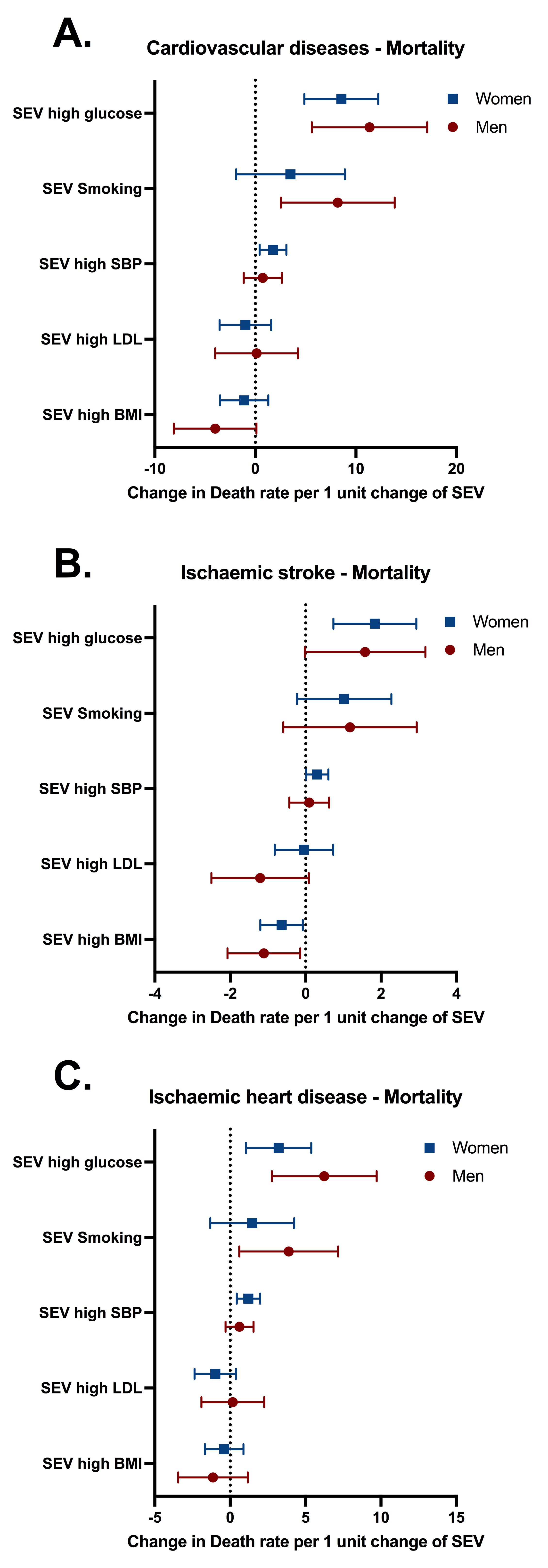

Supplement: S4 Fig — Unadjusted associations between SEV for risk factors and mortality due to (A) cardiovascular diseases, (B) ischaemic stroke, and (C) ischaemic heart disease for men and women in Brazil. Variables included in the model consisted of state and year fixed-effects and SEV of risk factors. Mortality and SEV were per 100,000 people. Estimates and 95% confidence intervals (CI) are provided in the figure. SBP: systolic blood pressure. LDL: low-density lipoprotein. BMI: body mass index. (TIFF) [file pone.0269549.s013.tiff]

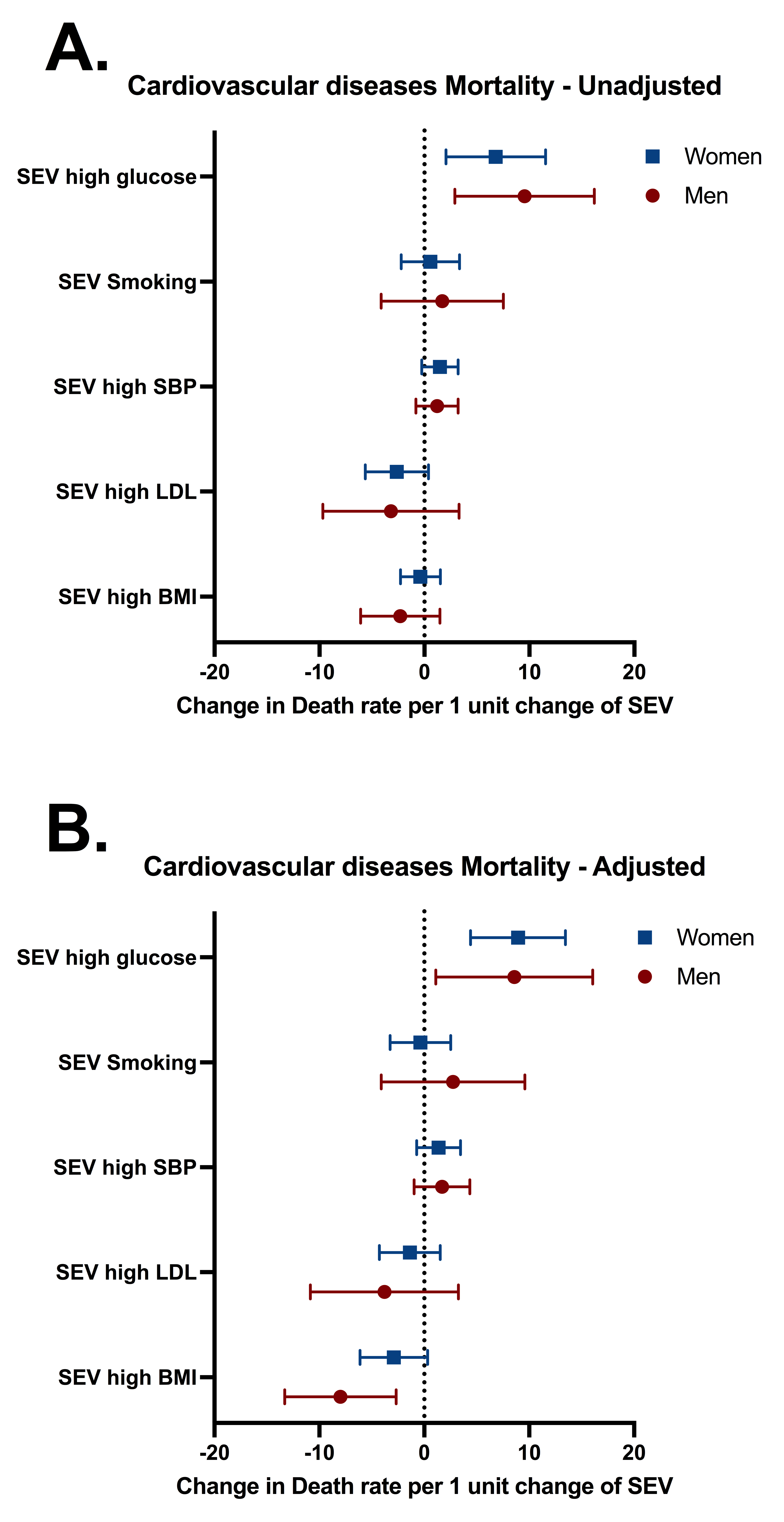

Supplement: S5 Fig — Risk factors were regressed with CVD mortality in different, independent models. The unadjusted model (A) consisted of state and year fixed-effects and SEV of risk factors. The adjusted model included: doctors per 1,000 habitants, hospital beds per 1,000 habitants, coverage of primary care, Bolsa Família transfer, GDP per capita, state and year fixed effects and SEV of risk factors. Mortality and SEV were per 100,000 people. Estimates and 95% confidence intervals (CI) are provided in the figure. SBP: systolic blood pressure. LDL: low-density lipoprotein. BMI: body mass index. (TIFF) [file pone.0269549.s014.tiff]
