# Supplementary material for: Analysing the impact of modifiable risk factors on cardiovascular disease mortality in Brazil
Source: PLoS One. 2022 Jun 22;17(6):e0269549. doi: 10.1371/journal.pone.0269549 (PMC9216570; doi:10.1371/journal.pone.0269549)
Supplement: Attachment — Submitted filename: Response to reviewers.docx [file pone.0269549.s016.docx]

Reviewer #1: Authors have examined the impact of changes in modifiable risk factors on CVD mortality in 26 Brazilian states from 2005 to 2017. As the results, authors have found Reduction in smoking was the risk factor that led to the prevention of highest number of CVD deaths, while hyperglycemia showed the most deleterious

association with CVD death. Manuscript is well written and results are very important.

However, It would be interesting to know what are the cancer incidence rate among these CVD patients and their deaths and how the cancer and its therapy impact the CVD deaths.

**Reply: We appreciate the reviewer's comments regarding our manuscript. After a thorough evaluation, we have concluded that your suggestion pertaining the incidence rate of cancer among CVD patients is an interesting idea that will be explored in a future study. We ask for the reviewer's understanding on this matter and thank you again for your time in evaluating our paper.**

Reviewer #2: The authors described the correlation between hyperglycaemia and CVD with disparities among different population gender groups in Brazil. It is interesting to note that women are more prone to diabetes and hyperglycaemia induced CVD than men. The study “Analysing the impact of modifiable risk factors on cardiovascular diseases mortality in Brazil" is a lucid read and the data presented through masses of statistics in the current research sets tone for the future directives with regard to CVD management in population in Brazil.

There are certain typographical errors in the writing which authors should improve.

With regard to availability of the data sharing of this research, it would be better if details where and how the data can be accessed and reused would be good.

**Reply: We thank the reviewer for taking the time to analyse our manuscript. After a thorough revision (both manually and using a grammar software named Grammarly), we have found several typos, as correctly pointed out by the reviewer. These typos were corrected in the revised version. Regarding data availability, we provide the URL of each database (supplementary table 1). Moreover, we are also uploading a spreadsheet containing all data (and models) used. To improve transparency, we now provide an R file with the codes used to generate the fixed-effects regression models. We appreciate the reviewer's time and hope that our changes have improved our manuscript.**
